# Assessment of the Contribution of BeiDou GEO, IGSO, and MEO Satellites to PPP in Asia–Pacific Region

**DOI:** 10.3390/s151229780

**Published:** 2015-12-01

**Authors:** Qile Zhao, Chen Wang, Jing Guo, Xianglin Liu

**Affiliations:** 1 GNSS Research Center, Wuhan University, No. 129 Luoyu Road, Wuhan 430079, China; zhaoql@whu.edu.cn (Q.Z.); chen.wang@whu.edu.cn (C.W.); 2 Fugro Intersite B.V., Leidschendam 2263 HW, The Netherlands; xianglin.liu@fugro.com

**Keywords:** BDS, precise point positioning, integer ambiguity resolution, convergence time, multi-GNSS

## Abstract

In contrast to the US Global Positioning System (GPS), the Russian Global Navigation Satellite System (GLONASS) and the European Galileo, the developing Chinese BeiDou satellite navigation system (BDS) consists of not only Medium Earth Orbit (MEO), but also Geostationary Orbit (GEO) as well as Inclined Geosynchronous Orbit (IGSO) satellites. In this study, the Precise Point Positioning (PPP) and PPP with Integer Ambiguity Resolution (IAR) are obtained. The contributions of these three different types of BDS satellites to PPP in Asia–Pacific region are assessed using data from selected 20 sites over more than four weeks. By using various PPP cases with different satellite combinations, in general, the largest contribution of BDS IGSO among the three kinds of BDS satellites to the reduction of convergence time and the improvement of positioning accuracy, particularly in the east direction, is identified. These PPP cases include static BDS only solutions and static/kinematic ambiguity-float and -fixed PPP with the combination of GPS and BDS. The statistical results demonstrate that the inclusion of BDS GEO and MEO satellites can improve the observation condition and result in better PPP performance as well. When combined with GPS, the contribution of BDS to the reduction of convergence time is, however, not as significant as that of GLONASS. As far as the positioning accuracy is concerned, GLONASS improves the accuracy in vertical component more than BDS does, whereas similar improvement in horizontal component can be achieved by inclusion of BDS IGSO and MEO as GLONASS.

## 1. Introduction

**Figure 1:**
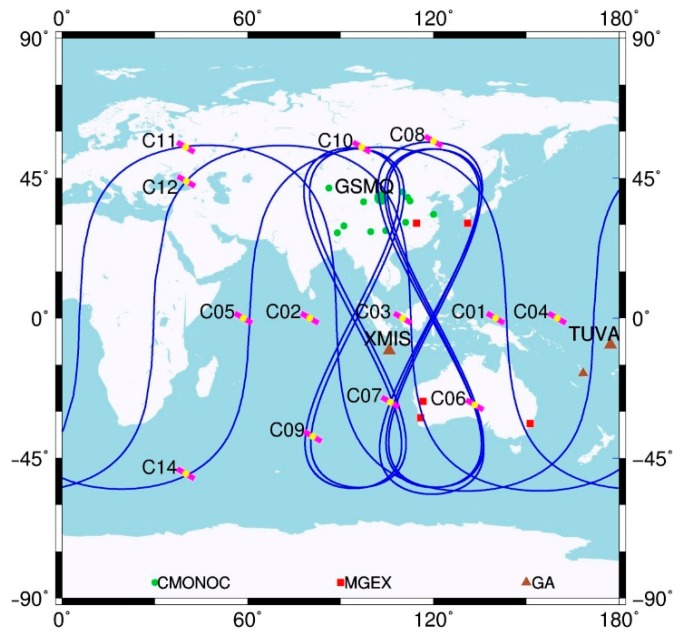
Ground footprint of BDS satellites and distribution of selected stations used in this study.

At the end of December 2012, the developing Chinese BeiDou satellite navigation system (BDS) was declared ready to provide Positioning, Navigation, and Timing service (PNT) in the Asia–Pacific region. The final system aims to be eventually a global navigation system like the US Global Positioning System (GPS) and the Russian Global Navigation Satellite System (GLONASS) as well as the European Galileo. The current constellation consists of five Geostationary Orbit (GEO), five Inclined Geosynchronous Orbit (IGSO), and four Medium Earth Orbit (MEO) satellites. The constellation is unique compared with the aforementioned GNSS systems forming by MEO satellites only. Figure 1 shows the ground tracks of BDS satellites. The IGSO satellites form two loops in the shape of figure eight with a mean longitude difference of roughly 30°, in order to provide an optimum coverage in China and its neighboring countries. The IGSO constellation is further complemented by five GEO satellites over the Indian and Pacific Ocean. Among the four MEO satellites, M05 (C13) does not work at present. The configuration of BDS constellation makes almost all IGSO and GEO satellites visible in Asia–Pacific region. With the additional MEO satellites, the BDS only Precise Point Positioning (PPP) with high accuracy is possible in this region.

Regarding PPP solutions using BDS only observations, there are some promising results. A ground network of 15 stations from BDS Experimental Tracking Network (BETN) established by Wuhan University are used to determine precise orbit and clock for three GEO and four IGSO satellites, and used these products for BDS only PPP [1]. Generally, the achieved RMSs (Root Mean Squares) of positioning differences with respect to (w.r.t.) the GPS-only solutions are better than 2 cm in horizontal and 7 cm in vertical for most static stations. The same level of positioning accuracy have been achieved with orbits and clocks determined with six stations [2]. Initial results of the orbits and clocks for four GEO, five IGSO, and two MEO satellites are determined using the BETN observations [3]. Thanks to better orbit and clock products and more available satellites, the RMSs of positioning differences between BDS only and GPS only solutions in static mode are around 1 cm in horizontal and better than 3 cm in vertical. The RMSs for kinematic solutions are within 1–2 cm in horizontal and 4–7 cm in vertical, respectively. Similar static and kinematic results have been reported by other researchers [4,5,6,7]. However, the BDS only PPP suffers from a long convergence time up to 60 min or even more to obtain centimeter-level positioning accuracy [6,7]. Fortunately, the signals of other existing or emergence GNSS systems can be used, and numerous studies have verified and assessed the possibility. For example, the results of [5] demonstrate that the BDS and GPS combined PPP solutions can not only improve the positioning accuracy but also accelerate the convergence speed, compared with the GPS only solutions.

However, these previous studies mainly investigated the accuracy and convergence time for PPP that could be achieved with all BDS satellites as a whole or their combination with other GNSS satellites. No publications analyze individually the contribution of different types of BDS satellites, more specifically, GEO, IGSO and MEO to the PPP solutions. Although the BDS only PPP results demonstrate the GEO satellites are vital to achieve better accuracy [2], particularly for the vertical component, the contributions of IGSO and MEO satellites are not analyzed further. As the geometry strength will be significantly reduced if any of the three types of satellites is not used, all of them obviously are essential for BDS only PPP. However, it is interesting to assess the contribution of these satellites when they are combined with each another, or combined with satellites of other GNSS system. This particularly meets the demand of the GNSS community that wants to assess the contributions of IGSO and GEO satellites as their altitude is much higher than that of the traditional MEO ones, and answers the question if these satellites can be used for PPP solutions.

In this study, we mainly aim at quantifying and analyzing different contributions of BDS GEO, IGSO, and MEO satellites to PPP solutions using different cases, e.g., static PPP using different types of BDS satellites, static and kinematic PPP using GPS and different types of BDS satellites. The possible contributions of BDS observations to shorten the convergence time and to the improvement in position accuracy, particularly in kinematic mode, are investigated. Importantly, these contributions to PPP Integer Ambiguity Resolution (IAR) of GPS satellites are also analyzed. The study focuses on the Asia–Pacific region as it makes more sense for the BDS satellites. Furthermore, those GPS and BDS combined static and kinematic PPP results are compared with that of GPS and GLONASS combined solutions to assess the contribution of BDS and GLONASS to GPS PPP. The three constellations combined solutions are also presented. The paper is organized as follows. In the strategy section, the observations, orbit and clock products as well as PPP and PPP IAR processing methodology are described. Numerical results with different PPP cases are presented and analyzed in results and analysis section. Finally, the study will be summarized in the last section.

## 2. Strategy

PPP is a powerful technique that provides centimeter or decimeter level positioning accuracy with a single receiver. No more corrections are needed for PPP except for satellite orbit and clock products [8], unless the integer ambiguity resolution is to be achieved. The accuracy of satellite orbit and clock products is crucial for PPP and has essential impact on both convergence time and positioning accuracy. As one of the analysis centers of IGS MGEX campaign [9], Wuhan University (WHU) determines orbits and clocks for GPS, GLONASS, Galileo, and BDS routinely. The strategy of precise orbit and clock determination for WHU MGEX products and their quality have been presented in [10,11]. The MGEX products of WHU (indicated as WUM) are used in this study. 

### 2.1. PPP

For PPP, the ionosphere-free combination of GPS, GLONASS or BDS measurements recorded in 30 s intervals from about 12 CMONOC (Crustal Movement Observation Network of China), five MGEX, and three GA (Geoscience Australia) stations observed during DOY (Day of Year) 252 to 282 in 2014 are used in this study (Figure 1). All of these stations are not used for both precise orbit determination and fractional cycle biases (FCB) generation (the latter will be described below). These stations are equipped with Trimble NetR9 receivers except for UNX3 that uses a Septentrio ASTERX3 receiver. The monthly average coordinates estimated by GPS only PPP are used as values of “ground-truth” for comparison later. For each station, the 24-h data set is divided into eight sessions. Considering the positioning errors introduced by orbit and clock interpolation, particular for BDS satellites, the first and last 3 h data are abandoned for processing. Hence, there are six sessions available starting from 3:00 a.m. to 9:00 p.m. in each day. For GPS only and combined PPP, the aforementioned data length is long enough to ensure the convergence of positioning. However, it may not be valid for BDS only PPP; in that case, the length of each session is set as 18 h instead of 3 h. Due to elevation dependent code errors of BDS IGSO and MEO satellites, the raw code observations are corrected using the piece-wise linear model developed in [12]. Considering no published receiver antenna Phase Center Offset (PCO) and Phase Center Variation (PCV) available for BDS signals for receiver antennas, the PCO and PCV corrections for GPS L1 and L2 are also used for BDS B1 and B2, respectively. Furthermore, the mask elevation is set to 10°, and the Square Root Information Filter (SRIF) is used as the estimation approach. The spectral density value for zenith tropospheric delay (ZTD) parameter is empirically set as 0.02 m^2^/s. The ambiguity parameters, inter-system biases (ISB), inter-frequency biases (IFB), and static position coordinates are considered as arc-dependent constants in the static mode. But in kinematic mode, the position coordinates are estimated in epoch wise. The initial standard deviations for raw code and phase observables of GPS, GLONASS as well as BDS IGSO and MEO are equally set to 2.0 m and 2.0 cm, respectively. However, we have down-weighted raw code and phase observations of BDS GEO to 4.0 m and 4.0 cm in this study. Table 1 summarizes the strategy used for PPP in detail, and Table 2 lists the PPP solutions used in this study and the corresponding abbreviations

**Table 1:** The strategy used for PPP.

Observable	Undifferenced Ionosphere-free combination of phases and code for GPS/GLONASS L1 and L2 as well as BDS B1 and B2
Elevation angle cutoff	10°
Sampling rate	30 s
Weight	Elevation-dependent weighting for the observations under 30° according to 1/2 sin(E)
Code biases	C1 observations are corrected to P1
Estimator	Square Root Information Filter (SRIF)
Troposphere	A prior model plus wet-component delay
	Mapping function: Vienna Mapping Function(VMF)
Satellite phase center correction	GPS, GLONASS from igs08.atx
	BDS: WHU estimated values [10,11]
Receiver phase center correction	GPS and GLONASS from igs08.atx
	Corrections for GPS L1 and L2 are also used for BDS B1 and B2
Initial standard deviations	2.0 m and 2.0 cm for raw code and phase of GPS, GLONASS as well as BDS IGSO and MEO
	4.0 m and 4.0 cm for raw code and phase of BDS GEO
Receiver clock	Estimated as epoch-wise white noise
ISB/IFB	Estimated as arc-dependent constants for each receiver or receiver-satellite pair
Coordinates	Constants in static mode
	Epoch-wise parameters in kinematic mode

**Table 2:** PPP solutions used in this study and their abbreviations.

PPP Solutions	Abbreviations
GPS only	GPS
BDS only	B
BDS GEO and IGSO	GI
BDS GEO and MEO	GM
BDS IGSO and MEO	IM
GPS plus BDS	G + B
GPS plus GLONASS	G + R
GPS plus BDS GEO	GPS + G
GPS plus BDS IGSO	GPS + I
GPS plus BDS MEO	GPS + M
GPS plus BDS GEO and IGSO	GPS + GI
GPS plus BDS GEO and MEO	GPS + GM
GPS plus BDS IGSO and MEO	GPS + IM
GPS, plus GLONASS, BDS IGSO and MEO	GPS + RIM

### 2.2. PPP Integer Ambiguity Resolution (IAR)

PPP IAR has the capacity of shortening the convergence time and improving accuracy. To do so, the so-called fractional cycle biases (FCB), *i.e.*, the satellite hardware biases, are needed. In this study, the approach proposed by [13] is used. In the reference side, the satellite FCB products of wide-lane and narrow-lane are estimated by utilizing the averaging of fractional parts of all pertinent wide-lane and narrow-lane ambiguity estimates derived from the Melbourne–Wübbena and ionosphere-free combination measurements, respectively. In the mobile side, the wide-lane and narrow lane FCB are applied to the corresponding ambiguities in order to remove the satellite hardware delay. As far as the receiver biases, single-differences between satellites are performed via selecting a reference satellite. The integer resolution strategy can be applied to the wide-lane firstly, and then to the narrow-lane ambiguities with being applied the fixed integers of wide-lane. Once the wide-lane and narrow-lane ambiguities have been fixed, the least-squares ambiguity decorrelation adjustment (LAMBDA) approach is used for ambiguity research, and the common used ratio test is used for ambiguity validation. The threshold of the ratio value is set as 3.0 [14]. In this contribution, we only made an attempt to fix the integer ambiguities for GPS phase observables. 

In this study, once the positioning error in three dimensions is less than one decimeter, and the accuracy criterion is met in the next 10 epochs; the positioning result is viewed as converged, and the convergence time is calculated from the beginning of positioning to the converged epoch. It should be mentioned that the positioning error is nothing but the difference between the solution and the ground-truth value.

## 3. Results and Analysis

In order to assess the contribution of BDS GEO, IGSO, and MEO satellites to PPP in Asia–Pacific region, the BDS only PPP in static as well as GPS and BDS combined PPP in both static and kinematic modes are performed. The corresponding convergence time and positioning errors are analyzed and compared in this section. Furthermore, GPS and GLONASS combined observations are also processed, and the corresponding ambiguity-float and -fixed PPP results are compared with that of GPS and BDS combined solutions in order to further compare the different contribution of BDS and GLONASS to GPS only PPP. In the meantime, the three constellations combined solutions are computed to demonstrate the level of precision and the convergence time achieved. In this section, the convergence time and position errors are used as the indicators of PPP results.

### 3.1. BDS Only PPP

Considering that exclusion of BDS GEO or IGSO measurements in data processing may not produce sensible kinematic PPP solutions due to the significant bad geometry, it is hard to assess the contribution of individual types of BDS satellites with kinematic BDS only PPP. Hence, the static PPP solutions are performed and investigated using at least two types of BDS satellite.

**Figure 2:**
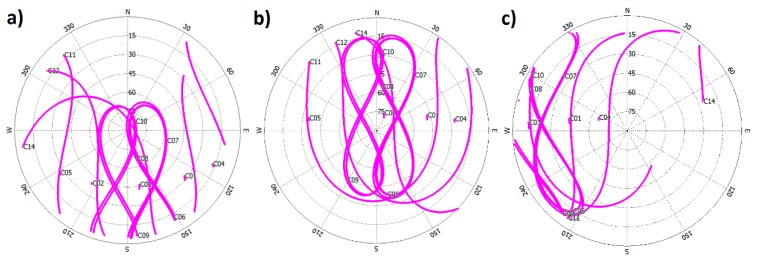
Sky-plots of (**a**) GSMQ; (**b**) XMIS; and (**c**) TUVA on DOY 252 in 2014.

All of the aforementioned sites are used for the result analysis. In the meanwhile, three selected stations named GSMQ, XMIS, and TUVA are, respectively, analyzed in detail, as they represent three typical observation conditions for BDS in Asia–Pacific region. The locations of these three stations are shown in Figure 1 together with other sites. Figure 2 illustrates the BDS satellites sky plots of the three sites. It can be seen that all of the GEO satellites are tracked continuously by GSMQ for the whole day, whereas IGSO satellites are visible for most time of one day (Figure 2a). This observation condition is similar for most of CMONOC stations. For XMIS (Figure 2b), all of GEO and IGSO satellites can be tracked continuously for the whole day. Compared with the other two sites, the observation condition for GEO and IGSO satellites is relatively poor in TUVA (Figure 2c). Specifically, only three GEO satellites could be tracked, and the elevation of C03 is rather low (about 15°). In addition, the five IGSO satellites in two separated orbital planes have almost overlapped trajectories and are located in the low elevation area, resulting in the worse observation condition. For BDS MEO satellites, the observation conditions are similar for the three sites, and the length of tracking session is about 8 h.

Table 3 lists the percentages of un-convergent sessions for the three selected sites individually and all sites as a whole in different BDS only static PPP solutions. The corresponding abbreviations can be seen in Table 2. Generally, the inclusion of BDS satellites in PPP could make more sessions to be converged due to the improved geometric strength. For PPP solutions using all BDS satellites in GSMQ and XMIS, which have good observation condition, all of the sessions are converged at least in 18 h, whereas the un-convergent percentage reaches only 3.3% for TUVA. The exclusion of GEO or MEO satellites in PPP solutions for GSMQ and XMIS will not change the percentage of un-convergence sessions, whereas the percentage increases significantly when the IGSO satellites are not used. Although not all sessions are converged in 18 h for TUVA when any type of satellites is not used for data processing, the GM solution still show the largest un-convergent percentage among GI, GM, and IM solutions. The similar phenomenon is also from the statistical results of all sites. Specifically, the un-convergent percentage is significantly high once IGSO satellites are not used in PPP. This indicates that the IGSO satellites make the greatest contribution to the reduction of convergence time for static BDS only PPP among these three kinds of satellites.

**Table 3:** Percentages of un-convergent sessions of static PPP solutions using different combinations of BDS satellites for the three selected individually and all 20 sites as a whole.

SITE	GI	GM	IM	B
GSMQ	0	12.6	0	0
XMIS	0	29.0	0	0
TUVA	25.8	45.2	16.1	3.3
ALL	2.0	21.8	1.0	0.3

**Figure 3:**
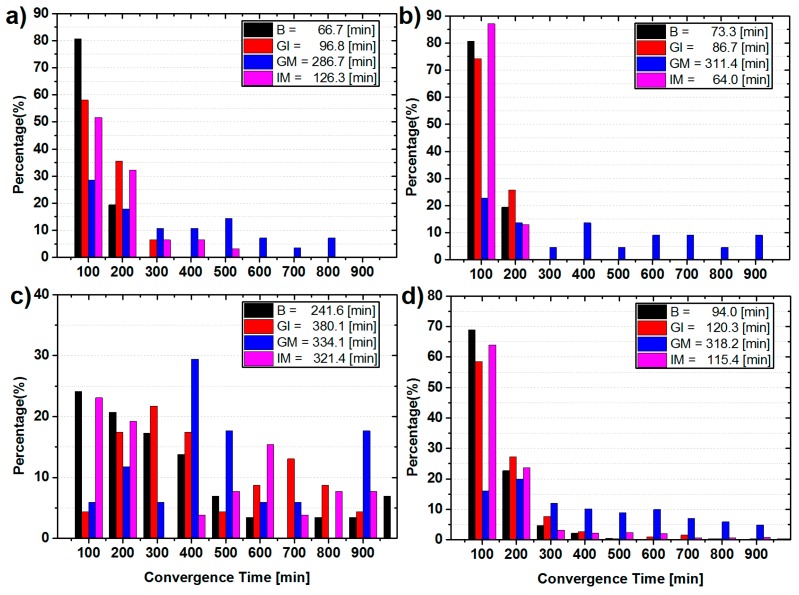
Statistics of convergence time in each 100 min and the averaged convergence time for the static BDS only PPP of (**a**) GSMQ; (**b**) XMIS; (**c**) TUVA; and (**d**) all 20 sites.

Furthermore, Figure 3 shows the statistical results of convergence time of the static BDS only PPP solutions for the three selected and all sites, respectively. By comparison of the convergence time of the solutions using any two types of satellites and using all BDS satellites, in general, from averaged convergence time for all 20 sites (Figure 3d), it can be seen that the inclusion of any kinds of BDS satellites can help to accelerate the convergence speed. For GSMQ (Figure 3a), the averaged convergence time is 66.7 min for PPP with all BDS satellites used, whereas it is 96.8 min for GI solutions, and 286.7 min and 126.3 min for GM and IM solutions, respectively. Similarly, the averaged convergence time of GM solutions for XMIS (Figure 3b) and all sites (Figure 3d) are the longest among the corresponding PPP solutions using any two types of satellites. This confirms that IGSO satellites make the largest improvement to the convergence time among the three types again. However, for TUVA (Figure 3c), the geometric condition of IGSO and GEO satellites is poor, hence, the contributions of IGSO satellites are weak, and the MEO satellites are most essential for PPP in this case.

The statistics of positioning errors for the convergent sessions are summarized in Table 4 for the selected three and all 20 sites, respectively. The standard derivation (STD) and RMS have been used for analysis in this study. For the static solutions, the positioning errors are the differences between the estimated coordinates in the last epoch of each session and the ground truth. Hence, the RMS is actually the root mean square of the positioning errors of all convergent sessions, and the difference between the RMS and STD is used to demonstrate whether there is noticeable positioning bias w.r.t the ground truth. For the kinematic solutions, the epoch-wise coordinates are determined, and the standard STD and RMS are computed with the coordinate differences from the convergent epoch to the last epoch in each session. Hence, the averaged values of STD and RMS from all sessions are used as the corresponding statistical values. 

**Table 4:** Position error statistics of convergent static PPP solutions using different combinations of BDS satellites for the three selected and all 20 sites.

Site	Solution	STD (cm)			RMS (cm)		
		E	N	U	E	N	U
GSMQ	B	0.56	0.53	1.32	0.74	0.75	1.94
	GI	0.68	0.66	1.69	0.71	0.80	2.54
	GM	2.17	1.16	2.74	2.24	1.34	2.80
	IM	0.59	0.32	1.28	0.78	0.58	2.07
XMIS	B	0.86	0.38	1.71	1.00	0.95	2.13
	GI	0.76	0.37	1.51	0.89	0.99	1.86
	GM	2.24	0.76	2.73	2.25	0.94	3.08
	IM	0.81	0.45	2.30	0.96	0.93	2.71
TUVA	B	1.89	1.24	2.65	1.90	1.26	2.66
	GI	2.48	2.87	4.74	2.68	2.95	6.12
	GM	2.85	0.86	3.99	2.94	1.01	4.05
	IM	2.21	0.97	2.72	2.26	1.13	2.72
ALL	B	0.78	0.57	1.82	1.05	0.76	2.38
	GI	1.10	0.70	2.12	1.29	0.91	2.81
	GM	2.41	0.99	3.02	2.60	1.10	3.35
	IM	0.84	0.49	1.86	1.11	0.70	2.59

In general, the results of PPP solutions with all BDS satellites are the best. For GSMQ, the positioning accuracy (RMS) is better than 1.0 cm and 2.0 cm in horizontal and vertical component, respectively. However, the positioning accuracy significantly decreases, particular in the east, once observables of IGSO satellites are not used for PPP solutions. Specifically, the degradation is about 1.50 cm, 0.59 cm, and 0.86 cm in the east, north, and vertical component, respectively. The overall statistics of all sites and site XMIS confirm this as well. This demonstrates the largest contribution of IGSO among three types of BDS satellites to the accuracy of static BDS only PPP, and the most contribution is in the east component. Considering that the positioning accuracy in east direction is highly related to the ambiguities, the continuous IGSO tracking by sites in Asia–Pacific region results in relative longer sessions of ambiguity parameters and sufficient geometric changes. Therefore, the corresponding ambiguities are relatively easier to be separated with other parameters, resulting in improvement of the positioning accuracy in the east direction. For TUVA, MEO satellites still play the most important role in PPP processing, and the noticeable degradation (about 3.46 cm) in vertical component has been observed when MEO observations are not used. The above conclusions are similar as that revealed by the analysis of convergence time in Figure 3.

### 3.2. Combination of GPS, GLONASS, and BDS for PPP Solutions

For the GPS based PPP solution, the addition of other satellites in processing will enhance the geometric strength, resulting in improvement of the positioning accuracy potentially. The performances of combined PPP are mainly guaranteed by GPS observations as to our investigation, and it will be shown later. In this section, we consider solutions of ten cases: GPS only, GPS + BDS, GPS + GLONASS, GPS + GLONASS and BeiDou IGSO, MEO, GPS + BDS GEO, GPS + BDS IGSO, GPS + BDS MEO, GPS + BDS GEO and IGSO, GPS + BDS GEO and MEO, and finally GPS + BDS IGSO and MEO. The corresponding abbreviations can be seen in Table 2. The performances of static and kinematic solutions for all sites are analyzed and compared. The analysis is still based on the convergence time and positioning accuracy.

#### 3.2.1. Ambiguity-Float PPP Solution

**Figure 4:**
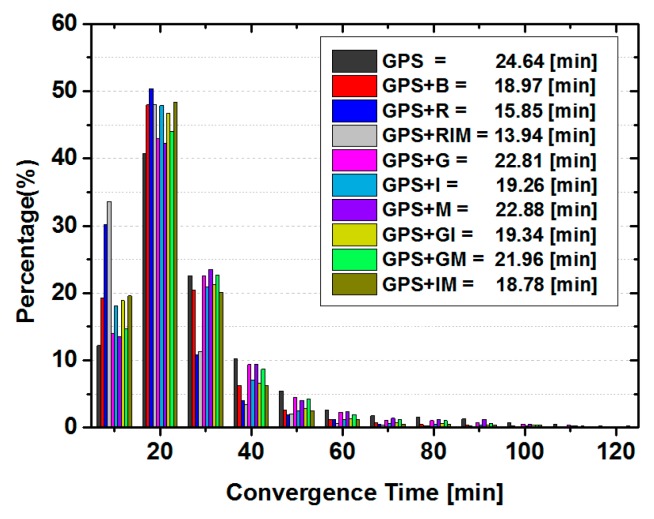
Statistics of convergence time in each 10 min and the averaged convergence time for the static ambiguity-float PPP solutions of all sites.

In this section, the impacts of BDS and GLONASS observations on the GPS ambiguity-float PPP are analyzed. Figure 4 demonstrates the percentage of convergence sessions in each 10 min period and the averaged convergence time for all static solutions computed from the aforementioned sites. As can be seen, different with that of BDS only static PPP, the convergence time is shorter, ranging from 0 to 50 min for most GPS only PPP solutions (about 95%). Only in occasional cases, when the solutions suffer from relatively few GPS satellites or bad data quality, the convergence time is longer than 50 min. Compared to the GPS only solution, the inclusion of any single type of BDS satellites accelerates the convergence speed to 22.81 min, 19.26 min and 22.88 min from 24.64 min for GPS + G, GPS + I, and GPS + M solutions, respectively. Again, IGSO satellites make the largest reduction to the convergence time. Compared the averaged convergence times of GPS + I solutions with that of GPS + GI, and GPS + B with GPS + IM solutions, it can be seen that the averaged convergence time become slightly longer after adding the GEO satellites. This indicates that GEO satellites might have the potential to slow the convergence speed of the GPS combined static PPP solutions once the IGSO observations are also used. However, the contamination is rather minor, at least with the strategy used in this contribution. 

On the other hand, once the GLONASS or BDS observations are combined with GPS, almost all PPP sessions are converged in 50 min thanks to better observation condition. The averaged convergence time is 24.64 min for GPS only PPP, while it is 18.97 min and 15.85 min for G + B and G + R solutions, respectively. However, only 1.91 min has been reduced when BDS IGSO and MEO are added in GPS and GLONASS combined PPP. Hence, those demonstrate that GLONASS makes larger reduction to the convergence time of GPS based combined PPP solutions than that of BDS, but the combination of the three GNSS systems could obtain the shortest convergence time among all these solutions.

Table 5 lists the statistics of positioning errors of all static PPP solutions computed from 20 sites. It can be seen that the positioning accuracy in each component is improved after adding either IGSO or MEO satellites to the PPP solutions. Although the accuracy in the east component is improved when GEO observables are combined with GPS data for PPP, it decreases slightly in other two directions. When we compare the position errors of GPS + I solutions with that of GPS + GI ones and GPS + B with GPS + IM solutions, it can be found that the positioning errors in each component become slightly larger after adding the GEO satellites. These are similar as that of convergence time. This indicates that GEO satellites also have the potential to contaminate the accuracy of the GPS and BDS IGSO combined static PPP solutions slightly. Compared the GPS + R solutions and GPS + I or GPS + IM solution, which are the best GPS and BDS combined solutions, it seems that the accuracy improvement in the vertical direction by inclusion of GLONASS observations is still larger than that of BDS. For other two components, BDS IGSO and MEO satellites make similar contribution to the accuracy as GLONASS satellites do. The GPS + RIM solution has the highest positioning accuracy among these ten solutions, while the accuracy improvement w.r.t GPS + R is mainly in the horizontal direction.

**Table 5:** Position error statistics of convergent static GPS ambiguity-float PPP solutions for all 20 sites.

TYPE	STD (cm)			RMS (cm)		
	E	N	U	E	N	U
GPS	1.32	0.63	1.69	1.64	0.81	1.98
GPS + B	1.11	0.60	1.63	1.39	0.78	1.97
GPS + R	1.01	0.56	1.55	1.29	0.76	1.87
GPS + RIM	0.84	0.53	1.32	1.16	0.73	1.69
GPS + G	1.31	0.66	1.75	1.60	0.83	2.02
GPS + I	0.98	0.57	1.57	1.29	0.77	1.93
GPS + M	1.26	0.61	1.67	1.57	0.80	1.96
GPS + GI	1.12	0.61	1.68	1.41	0.79	2.00
GPS + GM	1.30	0.64	1.72	1.58	0.83	2.01
GPS + IM	1.01	0.57	1.59	1.30	0.77	1.93

**Figure 5:**
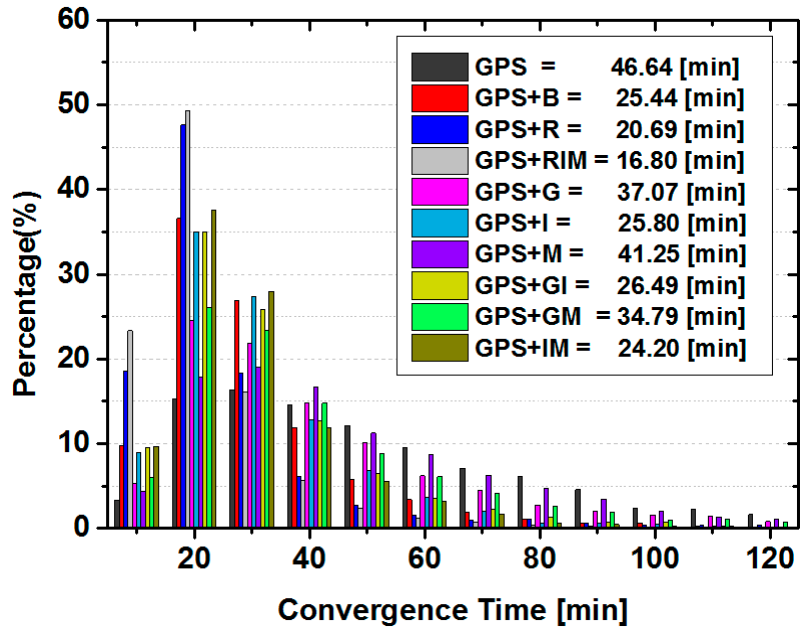
Statistics of convergence time in each 10 min and the averaged convergence time of the kinematic ambiguity-float PPP solutions for all sites.

For kinematic ambiguity-float PPP, the corresponding statistic results for convergence time are plotted in Figure 5. Due to the fact that position parameters are estimated in epoch-wise, the averaged convergence time of kinematic PPP is significantly longer than that of static PPP for both GPS only and all different combined solutions. Similar as that of static PPP, the inclusion of any single type of BDS satellites to GPS PPP (*i.e.*, GPS + I, GPS + M, GPS + G solutions) accelerates the convergence, and the GPS + I solutions converge fastest among three solutions. However, once GEO satellites are introduced to GPS + I or GPS + IM solutions, the averaged convergence time of GPS + GI and GPS + B solution slightly increase. The averaged convergence time is 46.64 min for GPS only PPP, whereas only 25.44 min, 20.69 min and 16.80 min for GPS + B, GPS + R, and GPS + RIM solutions, respectively. It is reduced significantly by 45.5%, 55.6% and 64.0%, thanks to the significant enhancement of geometric strength and redundancy by extra satellites in kinematic mode.

As for the statistics of positioning errors listed in Table 6, it can be seen that the BDS IGSO contributes most to the positioning accuracy among three kinds of BDS satellites by the comparison of GPS + G, GPS + I, and GPS + M solutions. Furthermore, the positioning accuracy could also be contaminated slightly by inclusion of GEO satellites to GPS and IGSO combined PPP solutions, *i.e.*, GPS + I and GPS + IM. Those results confirm the conclusion drawn from the static PPP solutions. The accuracy improvement in the vertical direction by combination of GLONASS and GPS is greater than that of BDS and GPS combined solution, whereas similar accuracy in horizontal direction has been achieved for GPS + R and GPS + IM solution. Finally, the GPS + RIM solution has the fastest convergence speed and highest positioning accuracy.

**Table 6:** Position error statistics of convergent kinematic ambiguity-float PPP solutions for all 20 sites.

TYPE	STD (cm)			RMS (cm)		
	E	N	U	E	N	U
GPS	1.87	1.08	2.59	2.38	1.27	3.00
GPS + B	1.17	0.77	2.20	1.66	0.99	2.71
GPS + R	1.03	0.69	1.86	1.44	0.89	2.22
GPS + RIM	0.86	0.61	1.71	1.23	0.83	2.13
GPS + G	1.67	0.96	2.52	2.21	1.16	2.90
GPS + I	1.09	0.78	2.01	1.53	0.98	2.51
GPS + M	1.72	1.01	2.45	2.23	1.19	2.87
GPS + GI	1.22	0.81	2.25	1.72	1.03	2.77
GPS + GM	1.61	0.93	2.46	2.15	1.12	2.84
GPS + IM	1.04	0.74	1.94	1.47	0.95	2.43

#### 3.2.2. Ambiguity-Fixed PPP Solutions

In this section, the impacts of BDS and GLONASS observations to aid GPS ambiguity-fixed PPP are analyzed. The solutions are produced in the same way as the ambiguity-float ones. It should be mentioned that the fixing ambiguity strategy is only applied to GPS satellites. In order to do so, the additional FCB corrections are applied to GPS satellites. We also computed static and kinematic PPP solutions in this section.

**Figure 6:**
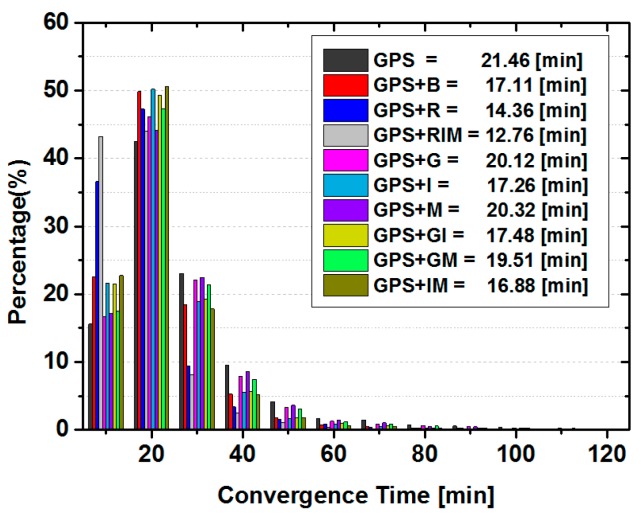
Distribution of convergence time in each 10 min and the averaged convergence time for the static GPS ambiguity-fixed PPP solutions for all sites.

Statistical results of the convergence time are plotted in Figure 6 for static PPP solutions. It can be seen that the convergence time of each solution is reduced when the GPS float ambiguities are fixed to the integers by comparison with the float results in Figure 4. The GEO satellites slightly slow the convergence of combined solutions when IGSO observations are also used. The convergence is accelerated more by introduction of GLONASS satellites than BDS ones.

The corresponding position errors are listed in Table 7. Different with the results of float-ambiguity solutions in Figure 4, the inclusion of any a single type of BDS observables does only improve the positioning accuracy in the east component. The other two components do not show significant differences. However, the positioning accuracy in all three directions is improved by adding GLONASS.

**Table 7:** Position error statistics of convergent static GPS ambiguity-fixed PPP solutions for all 20 sites.

TYPE	STD (cm)			RMS (cm)		
	E	N	U	E	N	U
GPS	0.82	0.54	1.52	1.16	0.75	1.80
GPS + B	0.72	0.53	1.53	1.06	0.74	1.88
GPS + R	0.63	0.51	1.28	1.02	0.73	1.59
GPS + RIM	0.56	0.49	1.25	0.94	0.72	1.52
GPS + G	0.79	0.56	1.57	1.15	0.77	1.85
GPS + I	0.60	0.52	1.47	0.98	0.74	1.83
GPS + M	0.74	0.54	1.50	1.10	0.75	1.80
GPS + GI	0.66	0.52	1.53	1.02	0.74	1.88
GPS + GM	0.80	0.55	1.55	1.14	0.76	1.84
GPS + IM	0.63	0.51	1.48	0.99	0.73	1.84

**Figure 7:**
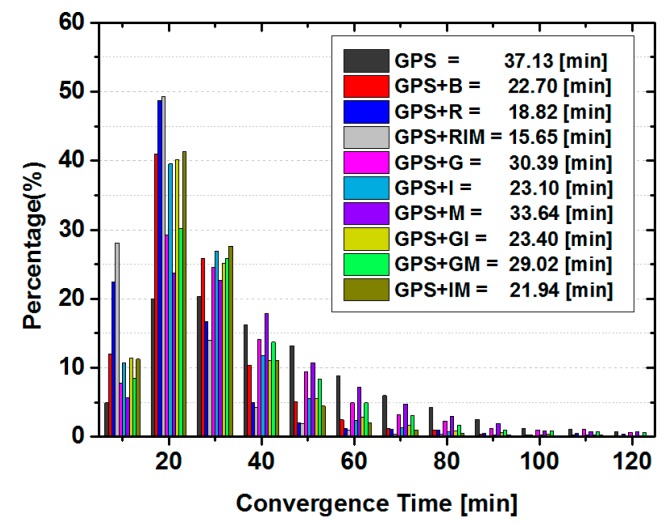
Distribution of convergence time in each 10 min and the averaged convergence time of the kinematic GPS ambiguity-fixed PPP solutions for all sites.

For the kinematic solutions, the statistical results of convergence time and positioning accuracy are shown in Figure 7 and Table 8, respectively. Compared with that of ambiguity-fixed static PPP solutions in Figure 6, the convergence time is longer and the accuracy is relatively low. However, the same conclusion for different contribution of different types of BDS satellites as well as BDS as a whole and GLONASS satellites to the convergence time and positioning accuracy can also be drawn.

**Table 8:** Position error statistics of convergent kinematic ambiguity-fixed PPP solutions for all 20 sites.

TYPE	STD (cm)			RMS (cm)		
	E	N	U	E	N	U
GPS	1.57	0.98	2.40	1.64	1.02	2.54
GPS + B	1.02	0.71	2.15	1.22	0.84	2.43
GPS + R	0.85	0.65	1.81	1.08	0.78	2.07
GPS + RIM	0.69	0.57	1.65	0.98	0.75	2.03
GPS + G	1.46	0.89	2.44	1.56	0.95	2.61
GPS + I	0.89	0.70	1.91	1.10	0.82	2.24
GPS + M	1.42	0.92	2.29	1.53	0.97	2.46
GPS + GI	1.06	0.74	2.20	1.26	0.86	2.49
GPS + GM	1.40	0.86	2.40	1.52	0.93	2.58
GPS + IM	0.84	0.67	1.86	1.07	0.80	2.20

## 4. Conclusions and Discussions

In this study, we use more than four weeks of data from 20 MGEX, BETS, and GA stations to assess the different contribution of BDS GEO, IGSO, MEO, and GLONASS observations to the convergence time and positioning accuracy in different cases. 

For BDS only static PPP, the PPP performances demonstrate that the IGSO satellites make the greatest contribution to the reduction of the convergence time and improvement of positioning accuracy of static PPP among the three kinds of BDS satellites for the sites with better IGSO tracking condition, whereas MEO observations are most essential for the sites with poor geometry.

Various numerical results show that the PPP by the combination of GPS and other GNSS observations can significantly reduce the convergence time for both ambiguity-float and -fixed PPP. However, the contribution of BDS to the reduction of convergence time is still not as large as that of GLONASS, this could be partly due to relatively lower quality of BDS orbit and clock products for the time being. Regarding the positioning accuracy of GPS based combined PPP solutions, GLONASS satellites could improve the accuracy in the vertical more than BDS ones, while in the horizontal component, the same accuracy improvement can be achieved by inclusion of either BDS IGSO/MEO or GLONASS satellites.

As to the contribution of different BDS satellites in general, the positioning performance is improved after adding any single type of BDS satellites to combine with the GPS ones. The IGSO satellites make the largest contribution to GPS based combined static and kinematic ambiguity-float or -fixed PPP. However, the statistical results demonstrate the inclusion of BeiDou GEO satellites could slightly degrade the position accuracy and slow the convergence only in the case that IGSO satellites are also used in combined solution. The negative contribution of GEO satellites in this case could be possibly caused by the multipath or potential satellite deduced systematic errors that are not optimally handled when combined with other observations [15,16,17]. In addition, the relative lower quality of orbits and clocks of GEO satellites may have some impacts that are difficult to be described by the stochastic model. In order to improve the situation, further investigation on refinement of stochastic model and better orbit/clock determination are needed. For the time being, the BDS GEO observations should be cautiously used in the Multi-GNSS constellation combined PPP solutions. However, we should realize that the BDS GEO still make positive contributions in some extreme observation situation (like site TUVA), which often happens in dynamic environment. It is noticed that one of the latest results demonstrated in [18] shows that the GEO BDS satellites slightly reduced convergence time and horizontal positioning errors. The results were achieved by simulated obstruction of signal tracking. This indicates the positive contribution of GEO BDS satellites can be achieved under challenge environment, which is again similar to the case of site TUVA.
